# Ai-Assisted Discovery of a Direct Physical Interaction Between a Venom Serpin from the Parasitoid Wasp *Liragathis javana* and a Host Serine Carboxypeptidase

**DOI:** 10.3390/toxins17120600

**Published:** 2025-12-16

**Authors:** Jiale Wang, Xunyuan Jiang, Zemiao Xiao, Xuemei Tang, Kai Wan

**Affiliations:** 1 Institute of Quality Standard and Monitoring Technology for Agro-Products, Guangdong Academy of Agricultural Sciences, Guangzhou 510640, China; jialewang@zju.edu.cn (J.W.);; 2 Guangdong Provincial Key Laboratory of Quality & Safety Risk Assessment for Agro-Products, Guangzhou 510640, China; 3 Key Laboratory of Testing and Evaluation for Agro-Product Safety and Quality, Ministry of Agriculture and Rural Affairs of the People’s Republic of China, Guangzhou 510640, China

**Keywords:** parasitoid wasp, *Liragathis javana*, venom, serpin, serine carboxypeptidase, host-parasitoid interaction, artificial intelligence, biological control

## Abstract

Parasitoid wasp venoms represent highly specialized biochemical arsenals that evolved to manipulate host physiology and ensure successful development of the parasitoid offspring. However, the molecular targets and mechanisms underlying this complex host modulation remain poorly understood. To address this, we employed an AI-driven discovery pipeline, integrating the sequence-based predictor D-SCRIPT with the structural modeler AlphaFold3, to characterize LjSPI-1, a venom serpin from *Liragathis javana*. This computational workflow highlighted a previously unreported candidate partner—a host serine carboxypeptidase (Chr09G02510). Crucially, we detected a direct physical interaction between these two proteins through both in vitro pull-down and in vivo yeast two-hybrid assays, supporting this AI-prioritized interaction under experimental conditions. Our study identifies a high-priority molecular pairing and demonstrates the utility of an AI-guided strategy for uncovering candidate targets of venom proteins. In addition, guided by the predicted biochemical role of Chr09G02510, we propose several plausible physiological hypotheses linking this interaction to host peptide metabolism and immune modulation. These hypotheses serve as a conceptual basis for future mechanistic and toxicological investigations.

## 1. Introduction

The parasitoid wasp *Liragathis javana* (Hymenoptera: Braconidae), formerly known as *Therophilus javanus* [1], has recently gained attention as a promising biological control agent of the legume pod borer, *Maruca vitrata* (Lepidoptera: Crambidae). This pest is one of the most destructive insects of cowpea and other legumes, with yield losses reaching up to 72% in affected regions [2,3]. In West Africa, intentional introductions of *L. javana* to Benin, Burkina Faso, and Niger demonstrated effective suppression of *M. vitrata* populations under field conditions [3,4,5,6]. Its recent detection in China’s mainland [7] further highlights its potential as a self-sustaining, host-specific component in integrated pest management (IPM) programs, especially in areas heavily dependent on chemical insecticides.

The biocontrol efficacy of *L. javana* largely depends on its venom, a complex mixture of proteins, peptides, and small molecules injected into *M. vitrata* larvae during oviposition. Venom proteins manipulate host physiology by suppressing immune defenses, disrupting development, inducing paralysis, and modulating metabolism, thereby ensuring parasitoid survival [8,9]. Among these components, serpins (serine protease inhibitors) represent a prominent class of venom effectors. In insects, serpins typically target serine protease cascades that regulate key immune processes such as hemocyte encapsulation and melanization, both of which are triggered by activation of the prophenoloxidase (proPO) system into active phenoloxidase (PO) [10,11,12,13]. Functionally characterized parasitoid venom serpins—for example, PpSerpin-1 isoforms from *Pteromalus puparum*, MmvSPN-1 and MmvSPN-2 from *Microplitis mediator*, and CvT-serpin6 from *Cotesia vestalis*—inhibit host hemolymph proteases involved in PPO activation and antimicrobial peptide induction, thereby suppressing host immunity [13,14,15,16].

Serpins share a conserved tertiary structure composed of three β-sheets and 7–9 α-helices, with the C-terminal reactive center loop (RCL) dictating protease specificity [17,18,19,20]. Their classical “suicide” inhibition mechanism involves cleavage of the RCL at the P1–P1′ bond, followed by its insertion into β-sheet A to form a covalent serpin–protease complex [12,21]. However, accumulating evidence indicates that the serpin can also mediate diverse non-canonical interactions beyond protease inhibition. For instance, Hsp47 (SERPINH1) functions as a collagen-specific molecular chaperone, and hormone carriers such as CBG (SERPINA6) and TBG (SERPINA7) exemplify additional regulatory roles [19,22,23]. In insects, serpins such as Spn4 from *Drosophila melanogaster* modulate neuropeptide and hormone precursor processing by targeting secretory-pathway proprotein convertases [24,25]. These findings underscore the structural and functional versatility of the serpin superfamily.

Despite these advances, the molecular basis for non-canonical serpin interactions in parasitoid venom remain poorly characterized, partly due to the limited availability of experimentally verified host targets. Recent developments in artificial intelligence-based protein interaction prediction and structure modeling, notably AlphaFold3 [26] and D-SCRIPT [27], now enables large-scale inference of protein–protein interactions directly from sequence data. When combined with biochemical validation, such AI-assisted workflows offer a powerful approach for uncovering previously unrecognized molecular relationships in host–parasitoid systems.

Here, we applied an AI-assisted discovery and validation framework to identify potential host targets for LjSPI-1, a venom serpin from *L. javana*. Using D-SCRIPT and AlphaFold3, we predicted and modeled its potential host partners from the *M. vitrata* proteome. Subsequent experimental assays, including pull-down and yeast two-hybrid, were employed to validate the predicted physical binding. Our analyses identified a previously uncharacterized serine carboxypeptidase as a candidate binding partner of LjSPI-1, providing experimental support for an AI-prioritized interaction. These findings highlight the power of AI-assisted approaches in defining novel molecular interactions and provide a specific, promising target for future studies on the evolutionary diversification and functional mechanisms of parasitoid venom serpins.

## 2. Results

### 2.1. Transcriptomic Profiling of L. javana Venom Transcripts

#### 2.1.1. Serpin Transcript DN708_c0_g2

Analysis of the venom-gland transcriptome of *L. javana* identified DN708_c0_g2, a serpin-like transcript, among the thirty most abundant venom-associated transcripts (Supplementary Table S2). This locus produced nine assembled isoforms with TPM values ranging from <10 to over 5000, indicating substantial variation in transcript abundance. However, none of the individual Trinity-assembled isoforms contained both a signal peptide (SP) and an RCL. Some isoforms had an SP but no RCL, while others carried an RCL but lacked the SP. Such mutually incomplete patterns are typical of de novo transcript assemblies of highly expressed venom genes and likely reflect assembly fragmentation rather than true biological splice variants.

To reconstruct a putative functional sequence, we combined the SP region from isoforms i9/i11 with the RCL-containing region from isoforms i2/i4 in silico. This full-length ORF was amplified from female *L. javana* cDNA and confirmed by Sanger sequencing. Mapping raw venom-gland reads to the validated ORF revealed continuous coverage across the entire 1194 bp coding region, with no uncovered bases (Supplementary Figure S1), confirming that the venom gland expresses a complete LjSPI-1 transcript despite the fragmented Trinity isoforms. The validated sequence was designated LjSPI-1 and selected for subsequent structural and functional characterization.

#### 2.1.2. Other Venom-Related or Highly Expressed Transcripts

In addition to DN708_c0_g2, several other transcripts were highly expressed in the venom gland of *L. javana* (Supplementary Table S2). These included multiple protease inhibitors, such as additional serpin-like sequences and neprilysin homologs, as well as annexin-like proteins and ribosomal components. Some transcripts showed high similarity to venom proteins reported from other parasitoid wasps, notably an 80 kDa venom protein from *Habrobracon hebetor* and a neprilysin-like protease from *Ceratina calcarata*. Furthermore, a subset of abundant transcripts encoded proteins of unknown function, indicating the presence of potentially novel venom effectors.

The diversity of these highly expressed transcripts suggests that *L. javana* venom comprises a complex mixture of proteolytic regulators, membrane-associated proteins, and uncharacterized factors. Such a repertoire is consistent with strategies used by parasitoids to manipulate host immune responses, metabolism, and development, and underscores the potential for *L. javana* venom to contain both conserved and unique molecular effectors.

### 2.2. Characterization of the Identified LjSPI-1

#### 2.2.1. Sequence Characteristics of LjSPI-1

The assembled full-length ORF of LjSPI-1 contained 1194 bp, encoding a predicted protein of 398 amino acids. SignalP analysis identified a signal peptide of 21 residues at the N-terminus, consistent with secretion into venom. Sequence analysis also revealed an RCL spanning residues 346–377, with the P17–P1 positions defined as Glu344 to Arg360, and the P1′–P14′ positions as Ser361 to Val374. The predicted P1 residue was Arg360, a basic amino acid often found in serpins that interact with trypsin-like proteases [20]. Together, these features indicate that LjSPI-1 shares structural characteristics with inhibitory serpins, although the precise protease specificity remains uncertain.

#### 2.2.2. Structural Features and Conserved Motifs

AlphaFold modeling predicted that LjSPI-1 adopts the canonical serpin fold, consisting of three β-sheets (A–C) and nine α-helices (hA–hI), together with the central RCL extending from β-sheet A. In addition, a short extra helix of three residues was predicted between hF and s3A, hereafter referred to as hF′, which represents a structural variation compared with classical serpins.

**Figure 1:**
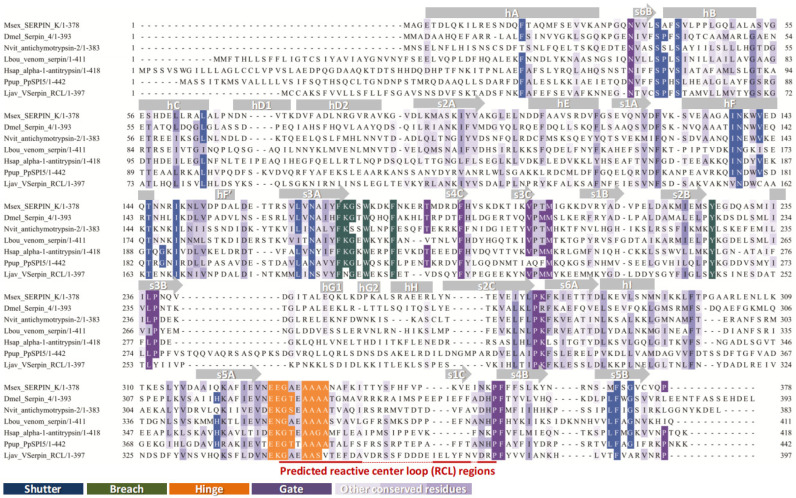
Multiple sequence alignment of LjSPI-1 with representative serpins. Alignment of LjSPI-1 with serpins from *Manduca sexta* (serpin 1K), *Homo sapiens* (α1-antitrypsin), *Pteromalus puparum* (PpSPI_5), and other insects. Conserved secondary structural elements and four key motifs (shutter, breach, hinge, and gate) are highlighted. The reactive center loop (RCL) is marked by a red line, and its intermittent sections reflect its inherent flexibility and sequence variability.

**Figure 2:**
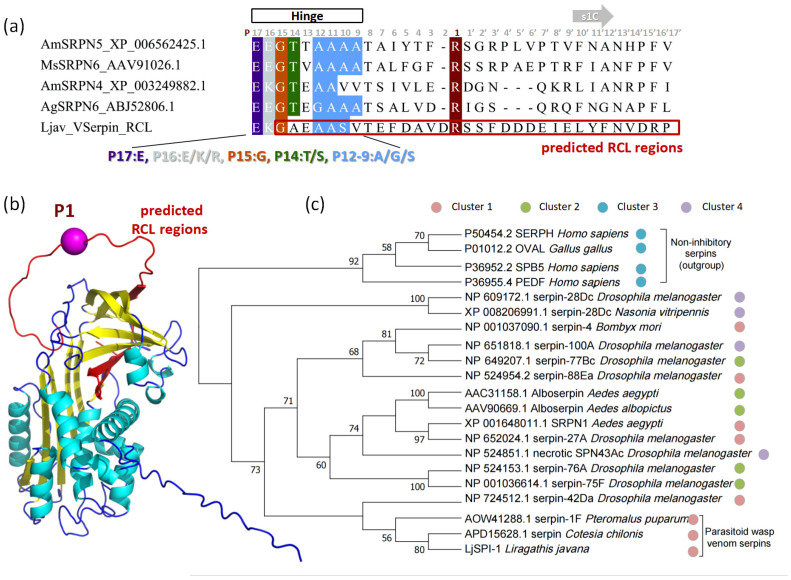
Structural and phylogenetic characterization of LjSPI-1. (**a**) Multiple sequence alignment of LjSPI-1 with insect serpins to identify the P1 position within the reactive center loop (RCL). Arg360 is highlighted as the P1 residue. (**b**) Predicted 3D structure of LjSPI-1 from AlphaFold3. α-Helices are shown in cyan, β-sheets in yellow, and loops/turns in blue. The RCL (residues 346–377) is highlighted in red, and the P1 residue (Arg360) is represented as a magenta sphere. (**c**) Maximum-likelihood phylogeny of LjSPI-1 and representative serpins with 1000 ultrafast bootstrap replicates and SH-aLRT tests. Bootstrap values ≥ 50% are shown at nodes. Colored circles beside each sequence label denote cluster assignments derived from ProtT5 embedding-based analysis, providing an orthogonal representation of functional similarity.

Based on MSA and structural comparison (Figure 1), conserved motifs typical of inhibitory serpins were identified. The shutter region corresponded to a hydrogen bond network centered on β-sheet A (s3A and s5A) and supported by surrounding elements, consistent with its role in regulating RCL insertion. The breach was located at the upper boundary of β-sheet A, representing the initial entry site for the RCL. The hinge is usually defined as the RCL segment P17–P9, providing flexibility for conformational change. Notably, the P14 residue in LjSPI-1 is Ala, in contrast to the strongly conserved Ser/Thr residues at this site in inhibitory serpins. In addition, the P9 position is occupied by Val instead of the typical small residues (A/G/S), which may further reduce hinge flexibility (Figure 2a). These atypical substitutions highlight structural divergence that could be linked to non-canonical functions.

The AlphaFold model (Figure 2b) was well supported by confidence scores, with an average per-residue pLDDT of ~84, most regions exceeding 90, and only loop regions dropping to ~20, indicating that the predicted fold is highly reliable except for flexible segments. In the structural model, the RCL (residues 346–377) was clearly exposed on the protein surface, consistent with its role in protease recognition. Interestingly, residues P10′–P13′ of the RCL were predicted to adopt a short β-strand rather than remaining as a flexible loop. In canonical inhibitory serpins, this downstream P′ segment is usually unstructured to permit efficient insertion of the cleaved loop into β-sheet A [28,29]. This unusual structural feature, together with atypical hinge substitutions, highlights potential divergence of LjSPI-1 from canonical inhibitory serpins.

#### 2.2.3. Phylogenetic and Embedding-Based Placement of LjSPI-1

A maximum-likelihood tree was constructed to examine the evolutionary placement of LjSPI-1 among representative serpins (Figure 2c). Non-inhibitory serpins (e.g., Hsp47, ovalbumin, PEDF, maspin) formed a basal cluster, confirming the expected separation from canonical inhibitory serpins. Within the inhibitory group, LjSPI-1 clustered with parasitoid venom serpins from *P. puparum* and *Cotesia chilonis* (bootstrap = 80). Its local relationship to *Drosophila* Spn4 (a synonym for Spn42Da) was weakly supported and was not interpreted as strict orthology. Other subclades were well resolved, including the Alboserpin/SRPN1/Spn27A/Spn43Ac group (bootstrap = 74) and the Spn75F/76A pair (bootstrap = 100). Taken together, these relationships are consistent with LjSPI-1 being part of the insect inhibitory serpin ensemble and evolutionarily closest to a subset of parasitoid venom and immune-related serpins, rather than to non-inhibitory serpins.

To complement the ML tree and provide an orthogonal, alignment-free representation of sequence similarity, we additionally performed ProtT5 embedding–based clustering using the same 21 serpin sequences. In both PCA- and UMAP-based projections (Supplementary Figure S2), LjSPI-1 fell into the same cluster as parasitoid venom serpins from *P. puparum* and *C. chilonis*, together with several immune-related inhibitory serpins from Lepidoptera and Diptera, whereas non-inhibitory vertebrate serpins formed a separate cluster. These embedding results therefore support the view that LjSPI-1 shares an inhibitory-type serpin scaffold and overall sequence features with venom/immune serpins, but—like the ML tree—do not specify which protease class it targets in vivo.

### 2.3. AI-Driven Prediction of a Novel Host Interaction Partner

#### 2.3.1. Candidate Target Identification by D-Script and Prioritization

To identify potential host targets for LjSPI-1, we screened the entire *M. vitrata* proteome using the sequence-based interaction predictor D-SCRIPT. This analysis yielded eight high-confidence candidates with prediction scores above 0.80 (Table S3). To prioritize these hits for further analysis, we applied a hierarchical filtering strategy based on biological relevance.

First, we focused on candidates annotated as peptidases or proteases, as these are the most plausible targets for a serpin and are known to be central to host manipulation. This criterion immediately highlighted four candidates: Chr06G02070 (a dipeptidyl peptidase), Chr09G02510 (a serine carboxypeptidase), and two alkaline phosphatases (Chr15G00770 and Chr15G00760). Other candidates, such as those involved in DNA replication (Chr18G03940) or copper transport (Chr24G01700, Chr18G04220), were considered less likely to be primary physiological targets for a venom serpin and were therefore deprioritized.

Second, we evaluated the peptidase candidates based on their physiological compatibility. Alkaline phosphatases exhibit optimal enzymatic activity in highly alkaline environments (typically pH > 8.0). However, parasitoid wasp venoms are characteristically acidic, with measured pH values ranging from approximately 4.0 to 6.5 in species such as *Ampulex compressa*, while host hemolymph generally maintains at near-neutral pH [30]. Given this discrepancy, it is physiologically implausible for a venom serpin to target a co-injected enzyme that would be largely inactive in both the venom reservoir and the host internal environment. Based on this strong biochemical rationale, the two alkaline phosphatases were excluded from further consideration. The remaining candidates, Chr06G02070 and Chr09G02510, emerged as the most compelling targets for downstream structural modeling.

#### 2.3.2. Structural Modeling by Alphafold3 and Binding Mode Analysis

**Figure 3:**
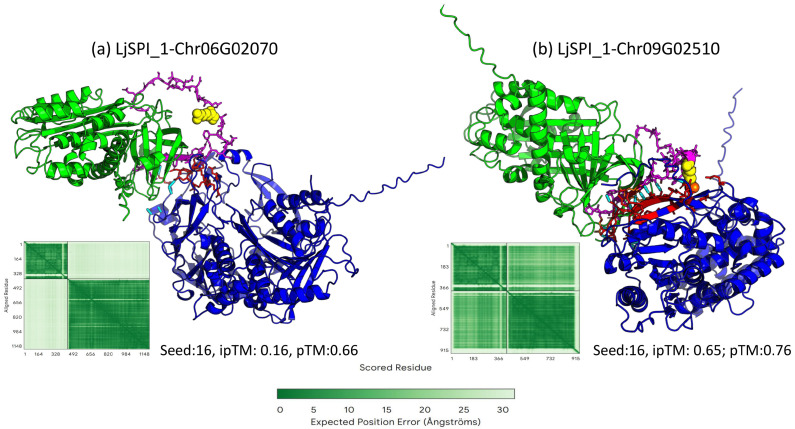
AlphaFold3-predicted binding interfaces and predicted aligned error (PAE) heatmaps for LjSPI-1 in complex with two candidate proteases. Predicted 3D structures show LjSPI-1 (green) in complex with candidate proteases (blue). The reactive center loop (RCL, magenta) and P1 residue (yellow sphere) were highlighted. Protease residues within 5 Å of the RCL are indicated in red, and predicted hydrophobic contacts are shown in orange. Cyan sticks represent hydrogen bonds at the modeled interface. In each panel, the inset in the lower left corner shows the corresponding PAE heatmap, where dark green indicates low predicted aligned error (high confidence) and lighter colors indicate increasing error (lower confidence). Panels correspond to: (**a**) LjSPI-1–Chr06G02070, (**b**) LjSPI-1–Chr09G02510.

To evaluate potential binding modes, we modeled the LjSPI-1 complexes with the two candidate proteases (Chr09G02510 and Chr06G02070) using AlphaFold3, generating 16 predictions per pair with different random seeds. The highest-ranking model by ipTM score for each complex is presented in Figure 3. Both proteins were individually predicted with very high confidence in all models (intra-chain PAE predominantly dark green, <5 Å). However, the inter-chain confidence differed dramatically between the two candidates.

For the LjSPI-1–Chr06G02070 complex, the top-ranked model exhibited a very low ipTM of 0.16 (pTM = 0.66). The inter-chain regions of the PAE plot were dominated by light green to white coloring, corresponding to expected positional errors predominantly >25 Å. This indicates that AlphaFold3 predicts no confidently defined binding interface, consistent with the peripheral and non-productive contacts observed in the structural model (Figure 3a). In contrast, the top-ranked LjSPI-1–Chr09G02510 model achieved an ipTM of 0.65 (pTM = 0.76; seed 16). Although the flexible RCL of LjSPI-1 exhibited the expected positional uncertainty, the inter-chain PAE displayed predominantly green values across much of the interface, suggesting moderate confidence in the overall docking orientation. In the rendered structure, several side-chain atoms of the LjSPI-1 P1 residue (yellow) overlapped with hydrophobic-contact selections and appeared partially orange, reflecting geometric proximity within the predicted model (Figure 3b).

Overall, these analyses suggest that AlphaFold3 provides strong support for the individual protein structures and moderate support for a plausible interaction orientation in the LjSPI-1-Chr09G02510 complex, whereas Chr06G02070 lacks structural or biological indications of interaction. Integrating AlphaFold3 modeling with biochemical considerations therefore identifies Chr09G02510 as the more likely physiological target of LjSPI-1.

### 2.4. Experimental Validation of a Physical Interaction Between LjSPI-1 and Chr09G02510

To experimentally test the top AI-predicted interaction, we selected Chr09G02510, an S28 serine carboxypeptidase, for in vitro and in vivo binding assays.

#### 2.4.1. Pull-Down Assay Demonstrates Direct In Vitro Binding

**Figure 4:**
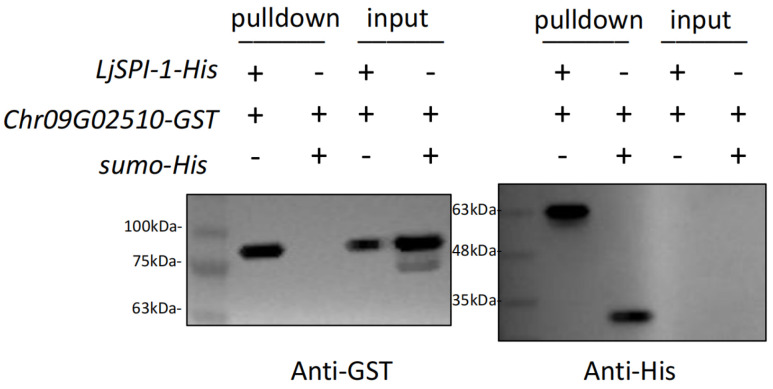
His-pulldown assay validating the interaction between LjSPI-1 and Chr09G02510. Purified LjSPI-1-His or control SUMO-His were immobilized on Ni-NTA magnetic beads and incubated with purified Chr09G02510-GST. “Pulldown” represents proteins eluted from beads after binding and washing. “Input” represents unbound proteins remaining in the supernatant after prey incubation. Anti-GST and anti-His Western blots detect prey and bait proteins, respectively. “+” indicates presence and “−” indicates absence of a protein in the reaction mixture.

To validate the predicted interaction, we performed His-tag pull-down assays using recombinant LjSPI-1-His as bait and the purified host protease Chr09G02510-GST as prey. Immunoblotting of the pull-down fraction with an anti-GST antibody revealed a clear band for Chr09G02510-GST when it was co-incubated with LjSPI-1-His (Figure 4, left panel). In contrast, this band was absent in the control reaction using an irrelevant SUMO-His protein as bait, demonstrating that the interaction is specific to LjSPI-1. Input controls confirmed the presence of the prey protein in the reaction mixtures prior to pull-down, and a parallel anti-His blot verified the robust immobilization of both His-tagged baits on the Ni-NTA beads (Figure 4, right panel).

#### 2.4.2. Yeast Two-Hybrid Assay Confirm In Vivo Interaction

**Figure 5:**
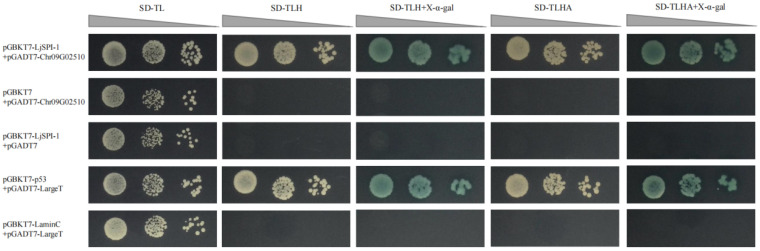
Yeast two-hybrid assay confirming the interaction between LjSPI-1 and Chr09G02510. Yeast strain AH109 was co-transformed with bait vector pGBKT7-LjSPI-1 and prey vector pGADT7-Chr09G02510. Positive (pGBKT7-p53 + pGADT7-largeT) and negative (pGBKT7-laminC + pGADT7-largeT) controls were included. Transformed clones were serially diluted (10^0^, 10^−1^, 10^−2^) and spotted onto SD/-Leu/-Trp (SD-TL), SD/-Leu/-Trp/-His (SD-TLH), SD-TLH + X-α-gal, SD/-Leu/-Trp/-His/-Ade (SD-TLHA), and SD-TLHA + X-α-gal plates. Triangles above each panel indicate serial 10-fold dilutions (10^0^, 10^−1^, 10^−2^) of yeast suspensions spotted onto the selective media.

To corroborate this interaction in a cellular context, we used a GAL4-based yeast two-hybrid system. We first confirmed that the LjSPI-1 bait construct did not auto-activate the reporter genes on its own (Supplementary Figure S2). In the interaction assay, yeast co-expressing LjSPI-1 (bait) and Chr09G02510 (prey) exhibited robust growth on high-stringency selective medium (SD/-Leu/-Trp/-His/-Ade) and developed strong blue coloration on plates supplemented with X-α-Gal, indicating a direct protein–protein interaction (Figure 5). The strength of this interaction was further supported by serial dilution spotting assays. All negative controls (e.g., LjSPI-1 with an empty prey vector) failed to grow under selective conditions, whereas the positive control (p53 + largeT) performed as expected. These results independently validate a direct and specific interaction between LjSPI-1 and Chr09G02510 in yeast.

Together, these data support the AI-guided identification of Chr09G02510 as a candidate binding partner of LjSPI-1 and demonstrate direct interaction under the tested conditions. However, enzyme assays and additional specificity controls, such as testing unrelated proteases or LjSPI-1 mutants, are still required to establish the functional relevance and specificity of this interaction.

## 3. Discussion

In this study, we employed an AI-driven discovery pipeline to explore the host interactome of a venom serpin, LjSPI-1, from the parasitoid wasp *L. javana*. This approach prioritized a previously unrecognized host candidate, the serine carboxypeptidase Chr09G02510, and our experiments provide evidence that LjSPI-1 and Chr09G02510 can form a direct physical complex under the tested conditions. The detection of this interaction by both in vitro pull-down and in vivo yeast two-hybrid assays offers an experimentally supported candidate host binding partner for a parasitoid venom serpin and expands current thinking beyond the canonical view that venom serpins primarily target serine protease cascades such as the prophenoloxidase system [15,16].

Our integrative structural and sequence analyses collectively support placing LjSPI-1 within an insect inhibitory serpin lineage, in close proximity to parasitoid venom serpins. Both the maximum-likelihood phylogeny and the ProtT5-based embedding analysis place LjSPI-1 alongside well-characterized inhibitory serpins, indicating that its overall fold, conserved motifs, and evolutionary context are consistent with an inhibitory-type serpin scaffold. At the same time, several features distinguish LjSPI-1 from classical suicide inhibitors. Its RCL carries non-canonical substitutions at key hinge positions (e.g., P14 = Ala, P9 = Val), and additional structural elements, such as a short hF′ helix and a predicted β-strand within the P′ segment, may impede the conformational flexibility required for standard suicide inhibition [31,32,33,34,35,36,37]. These deviations raise the possibility that LjSPI-1 may employ a modified inhibitory mechanism or engage proteases in a non-covalent regulatory manner. Consistent with this interpretation, the AlphaFold3 complex models provide supportive evidence for a potential interaction between LjSPI-1 and Chr09G02510. AlphaFold3 predicts the monomeric structures of both proteins, and across multiple seeds the protease is consistently positioned in proximity to the RCL region of the LjSPI-1, where several P1 side-chain atoms fall within the hydrophobic-contact selection. However, the predicted inter-chain confidence values fall into an intermediate range, indicating that while the model supports the possibility of interaction, the precise geometry remains incompletely resolved. This “gray-zone” confidence is expected for systems involving flexible RCL-mediated contacts and does not preclude biologically meaningful association; rather, it highlights the need for experimental follow-up.

A crucial aspect of this study is the acknowledgment of its current limitations regarding the functional consequences of this interaction. Although we obtained soluble recombinant Chr09G02510 from a prokaryotic expression system, our current assay conditions have not yet detected reproducible enzymatic activity or revealed whether LjSPI-1 modulates its catalysis in vitro. Moreover, His-tag pull-down and yeast two-hybrid assays, while widely used to demonstrate direct protein–protein contacts, are intrinsically susceptible to false positives, particularly with heterologously expressed proteins. Our interaction data should therefore be viewed as evidence for a specific physical association under the conditions tested, supported by two complementary assay platforms and by structural and evolutionary context, rather than as definitive proof of a fully validated physiological serpin–protease pair. Importantly, these caveats do not diminish the main contribution of this study, which is to establish an AI-guided pipeline that converges on a non-canonical serpin–carboxypeptidase pairing and to identify a high-priority candidate interaction for future functional work. This limitation must be addressed in future studies to conclusively validate the functional implications of the interaction.

Nevertheless, the identification of Chr09G02510 as a candidate binding partner for LjSPI-1 allows us to formulate testable hypotheses about how this interaction might contribute to host manipulation. Chr09G02510 belongs to the S28 serine carboxypeptidase family and is most closely related to the dipeptidyl peptidase II (DPP II) encoded by K12H4.7 in *Ostrinia furnacalis* (identity 72.56%, E-value < 1 × 10^−5^), suggesting that it may represent a DPP7/DPPII-like S28 exopeptidase that removes N-terminal dipeptides from bioactive peptides and thereby regulates their turnover. If Chr09G02510 is indeed an active DPP7/DPPII-like S28 protease in vivo, at least two non-exclusive consequences of its interaction with LjSPI-1 can be envisaged. First, host neuropeptide turnover and endocrine signaling could be affected. DPP7/DPPII-like S28 proteases have been implicated in the degradation of bioactive peptides, including neuropeptides and peptide hormones, thereby shaping their half-lives and signaling intensity [38,39,40,41]. By binding to Chr09G02510, LjSPI-1 could in principle alter the rate at which such peptides are cleared, either accelerating their degradation or stabilizing them against proteolysis. Either outcome would be expected to perturb endocrine homeostasis and neuromodulatory pathways, with potential consequences for host locomotion and development. At present, these possibilities remain speculative and will require direct measurements of Chr09G02510 activity and peptide turnover in the presence of LjSPI-1.

Second, LjSPI-1 may influence host immune function. The antimicrobial peptide (AMP) response is a critical determinant of successful parasitism, and most characterized venom serpins act by suppressing AMP production through inhibition of upstream serine protease cascades; for example, Spn43Ac in *Drosophila* inhibits Toll pathway proteases, and similar effects have been reported for serpins in *B. mori* [42,43,44,45,46]. If Chr09G02510 contributes, directly or indirectly, to the degradation or processing of AMPs or related immune peptides in *M. vitrata*, binding by LjSPI-1 could shift the balance between AMP production and clearance, thereby modulating the magnitude or duration of the immune response. This proposed role of a venom serpin in controlling AMP turnover, rather than AMP production, represents an attractive working hypothesis that will need to be tested by future functional studies.

Taken together, these hypotheses suggest that LjSPI-1 may act on host proteases central to peptide metabolism, with the potential to influence both immune and neuromodulatory pathways to facilitate successful parasitism. However, they remain speculative until Chr09G02510’s enzymatic activity and its modulation by LjSPI-1 are directly demonstrated.

## 4. Conclusions

In summary, our study demonstrates the utility of an AI-powered approach for rapidly identifying candidate molecular interactions in complex biological systems. We have uncovered and experimentally supported a previously unrecognized physical link between a parasitoid venom serpin and a host carboxypeptidase. Rather than defining a fully validated functional mechanism, our findings establish a robust candidate molecular pairing and provide a foundation for future work aimed at determining its biological consequences and specificity. This work contributes to our understanding the intricate co-evolutionary arms race between hosts and parasitoids and may offer novel targets for future pest management strategies.

## 5. Materials and Methods

### 5.1. Insect Collection and Rearing

Samples of the parasitoid wasp *Liragathis javana* and its host *Maruca vitrata* were collected in Guangzhou, China (23°18′ N, 113°35′ E). Larvae of *M. vitrata* were maintained under controlled laboratory conditions (L:D = 16:8, 25 ± 1 °C, 60% relative humidity). Each larva was housed individually in disposable culture dishes until parasitoid larvae emerged. An artificial diet was provided, consisting of soybean powder, red bean powder, agar, wheat germ, glucose, casein, cellulose, ascorbic acid, vitamins, sorbic acid, methylparaben, cholesterol, and β-sitosterol. Adult parasitoids were reared on 20% (*v*/*v*) honey-water solution.

### 5.2. Transcriptome and Sequence Analysis

Venom gland transcriptome data of *L. javana* were obtained from the NCBI SRA database (SRR14710222), consisting of 145.3 million paired-end reads (2 × 125 bp) generated on an Illumina HiSeq 2500 platform. For this dataset, total RNA was extracted from 30 venom glands, enriched for poly(A) + mRNA, and sequenced by Genewiz (France) under BioProject PRJNA734452 (BioSample SAMN19492040). Raw reads were trimmed with Trimmomatic (v0.39), evaluated with FastQC (v0.11.9), assembled using Trinity (v2.8.5), and quantified in TPM (Transcripts Per Million) using Salmon (v1.5.2) in quasi-mapping mode. Functional annotation was performed against the NCBI non-redundant protein database (NR) using the OmicShare platform (https://www.omicshare.com/; accessed on 10 April 2024). For experimental validation, *L. javana* samples were independently collected in Guangzhou, China (described in Section 2.1). Total RNA was extracted from these samples to amplify the full-length LjSPI-1 ORF, which was subsequently verified by Sanger sequencing. First-strand cDNA was synthesized using the SynScript^®^ III RT SuperMix for qPCR (TSK314S, Tsingke Biotechnology, Ezhou, China).

### 5.3. Sequence Validation and Characterization

The full-length cDNA of LjSPI-1 was amplified from *L. javana* venom gland RNA and verified by Sanger sequencing (Tsingke Biotechnology, Wuhan, China). Signal peptides were predicted using SignalP 6.0, and open reading frames (ORFs) were identified with ORF Finder (NCBI). Conserved motifs and domains were annotated using HMMER (v3.3.2) with the Pfam database. Basic protein properties, including molecular weight and isoelectric point, were calculated using the ProtParam module of Biopython (v1.81). Multiple sequence alignment (MSA) with representative insect serpins was generated with Clustal Omega (https://www.ebi.ac.uk/jdispatcher/msa; accessed on 30 May 2024), and sequence logos were visualized with Jalview (v2.11.50). To evaluate whether fragmented Trinity isoforms represent assembly artifacts, raw venom-gland RNA-seq reads were mapped to the Sanger-validated LjSPI-1 ORF. Bowtie2 (v2.5.1) was used to index and align clean paired-end reads to the LjSPI-1 cDNA, and per-base coverage was obtained with samtools. The coverage plot was generated using a custom Python (v3.8.10) script with matplotlib.

### 5.4. Structural Modeling and Phylogenetic Analysis

To prepare for AI-assisted interaction prediction, we first modeled the structure of LjSPI-1 using AlphaFold3 (https://www.alphafoldserver.com; accessed on 6 June 2024) and established its evolutionary context. Secondary structural elements and key functional motifs (e.g., shutter, breach, hinge, gate) were annotated based on both the AlphaFold model and multiple sequence alignment (MSA). Homologous serpin sequences were retrieved from GenBank (https://www.ncbi.nlm.nih.gov/genbank; accessed on 3 June 2024) and aligned with MAFFT (v7.505). Poorly aligned and gap-rich regions were removed with trimAl (v1.4) to retain the conserved serpin core. Phylogenetic analysis was conducted with IQ-TREE (v1.6.12), and the best-fit substitution model identified by ModelFinder was LG + G4. Branch support was evaluated using 1000 ultrafast bootstrap replicates and SH-aLRT tests. The list of representative sequences used for alignments and tree construction is provided in Supplementary Table S1.

### 5.5. Interaction Protein Prediction and Structural Modeling

An AI-assisted workflow integrating sequence-based and structure-based prediction was applied to identify potential host targets of LjSPI-1. Potential host proteases encoded by the *M. vitrata* genome (GenBank assembly GCA_039566165.1) [47] were screened for potential interactions with LjSPI-1 using D-SCRIPT (v0.2) [27]. Predictions were performed against the complete predicted proteome of *M. vitrata*, and candidates were filtered based on interaction probability scores. All candidates were annotated with HMMER (v3.3.2) against the Pfam database. Prioritized candidates were modeled in complex with LjSPI-1 using AlphaFold3 with default parameters. PyMOL (v2.5.5) was used for structural visualization, highlighting secondary structure elements and potential binding interfaces.

### 5.6. Embedding-Based Serpin Clustering Analysis Using ProtT5

To complement the ML-based phylogeny, we performed an alignment-free clustering analysis using protein language model embeddings. All 21 representative serpin sequences were embedded using the ProtT5-XL model (Rostlab/prot_t5_xl_half_uniref50-enc). Each sequence was converted into a 1024-dimensional vector by mean pooling of the final hidden states. The resulting embeddings were standardized and grouped using k-means clustering (k = 4). For visualization, dimensionality reduction was conducted using principal component analysis (PCA) and UMAP (n_neighbors = 15). These embedding-derived clusters were subsequently mapped onto the ML phylogeny (Figure 2c).

### 5.7. Protein Expression, Purification, and Pull-Down Assay

To validate the interaction predicted by our AI-assisted pipeline, we first performed pull-down assays with recombinant LjSPI-1 and Chr09G02510. The LjSPI-1 coding sequence (without the signal peptide) was cloned into pET-28a-SUMO (NcoI/XhoI) and expressed in *Escherichia coli* BL21(DE3) as an N-terminal His-tagged SUMO fusion. The prioritized host protease candidate, Chr09G02510, coding sequence (without the signal peptide) was cloned into pGEX-6P-1 (BamHI/EcoRI) and expressed as an N-terminal GST fusion. Recombinant proteins were expressed in LB medium and induced at OD_600_ ≈ 0.5 with 0.2 mM IPTG, followed by overnight incubation at 16 °C. Proteins were purified using Ni-NTA agarose or glutathione Sepharose (Smart-Lifesciences Biotechnology Co., Ltd., Changzhou, China), and purity was assessed by SDS-PAGE. For pull-down assays, His-tagged LjSPI-1 or His-SUMO control (0.3 µg) was immobilized on Ni-NTA magnetic beads (30 µL; Qiagen, Hilden, Germany) for 3 h at 4 °C. Beads were washed three times with PBS and incubated overnight at 4 °C with GST-tagged Chr09G02510 (0.3 µg). After additional washes, bound proteins were eluted with 100 µL PBS containing 500 mM imidazole and analyzed by SDS-PAGE and Western blotting with anti-His and anti-GST antibodies, followed by HRP-conjugated secondary detection and chemiluminescence (SuperSignal™ West Pico, Thermo Scientific, Waltham, MA, USA).

### 5.8. Yeast Two-Hybrid Assay

To corroborate the AI-predicted LjSPI-1–Chr09G02510 interaction in a cellular system, a GAL4-based yeast two-hybrid assay was conducted. LjSPI-1 and Chr09G02510 coding sequences (without signal peptides) were cloned into the pGBKT7 and pGADT7 vectors, respectively, and co-transformed into Saccharomyces cerevisiae AH109. Transformants were selected on SD/-Leu/-Trp (DDO) medium. Protein–protein interactions were screened on SD/-Leu/-Trp/-His/-Ade (QDO) plates in the presence or absence of X-α-Gal. Positive (p53 + largeT) and negative (laminC + largeT) controls were included, and yeast suspensions were serially diluted (10^0^ to 10^−2^) and spotted onto selection plates. Interaction was confirmed by yeast growth and blue coloration after incubation at 30 °C.

## Data Availability

All data supporting the findings of this study are included in the article and its Supplementary Materials. Transcriptome data are publicly available at NCBI SRA (accession number SRR14710222). Host genome data are available under NCBI Genome assembly ASM3956616v1, GenBank assembly GCA_039566165.1, and WGS project JBBMWA01, submitted by the Guangdong Academy of Agricultural Sciences. Additional data will be made available upon reasonable request to the corresponding author.

## Supplementary Materials

The following supporting information can be downloaded at: https://www.mdpi.com/article/10.3390/toxins17120600/s1, Figure S1. Per-base RNA-seq coverage across the validated LjSPI-1 ORF. Figure S2. Embedding-based clustering of 21 representative serpin proteins using ProtT5. Figure S3: Autoactivation control of the bait construct pGBKT7-LjSPI-1 in yeast two-hybrid assay; Table S1: Serpin sequences from other species used in this study; Table S2: Detailed information on the top 30 highly expressed genes and their isoforms in the venom gland of *Liragathis javana*; Table S3: Candidate LjSPI-1-interacting proteins predicted by D-Script with scores > 0.80, Pfam domain annotation, and functional description.
