# Supplementary material for: Ai-Assisted Discovery of a Direct Physical Interaction Between a Venom Serpin from the Parasitoid Wasp Liragathis javana and a Host Serine Carboxypeptidase
Source: Toxins (Basel). 2025 Dec 16;17(12):600. doi: 10.3390/toxins17120600 (PMC12737784; doi:10.3390/toxins17120600)
Supplement: Supplementary file 1 [file toxins-17-00600-s001.zip › Supplementary figures-R1.pdf]

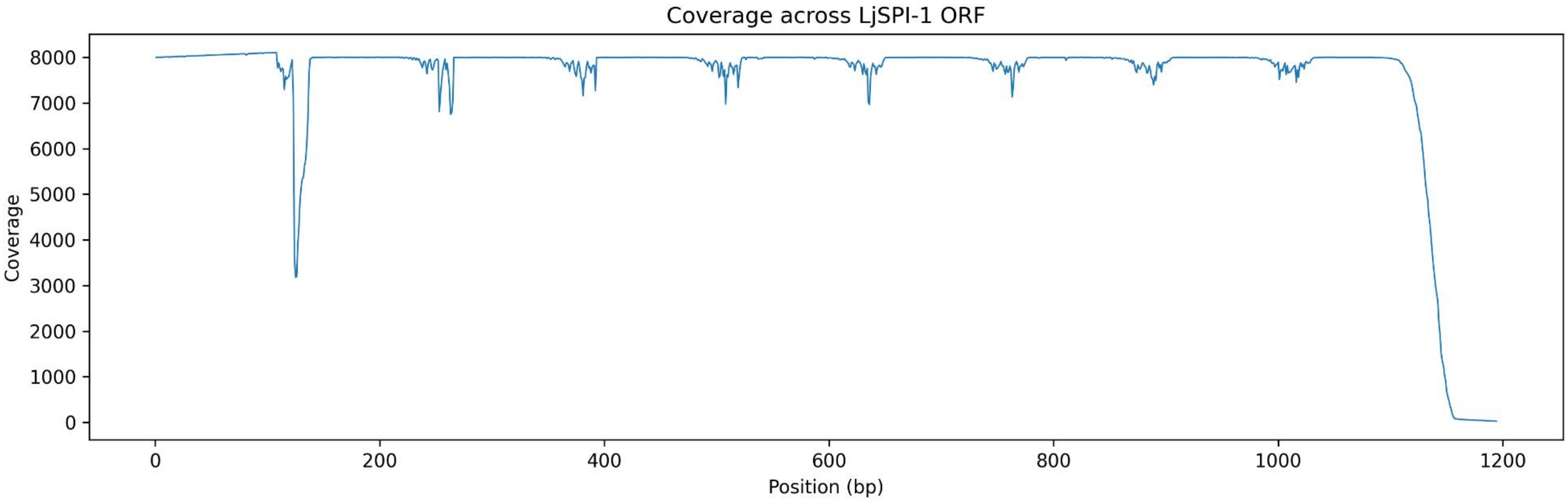

Figure S1. Per-base RNA-seq coverage across the validated LjSPI-1 ORF. Coverage is continuous across the entire 1,194 bp coding region, confirming that the full-length transcript is supported by raw reads despite fragmentation of Trinity-assembled isoforms.

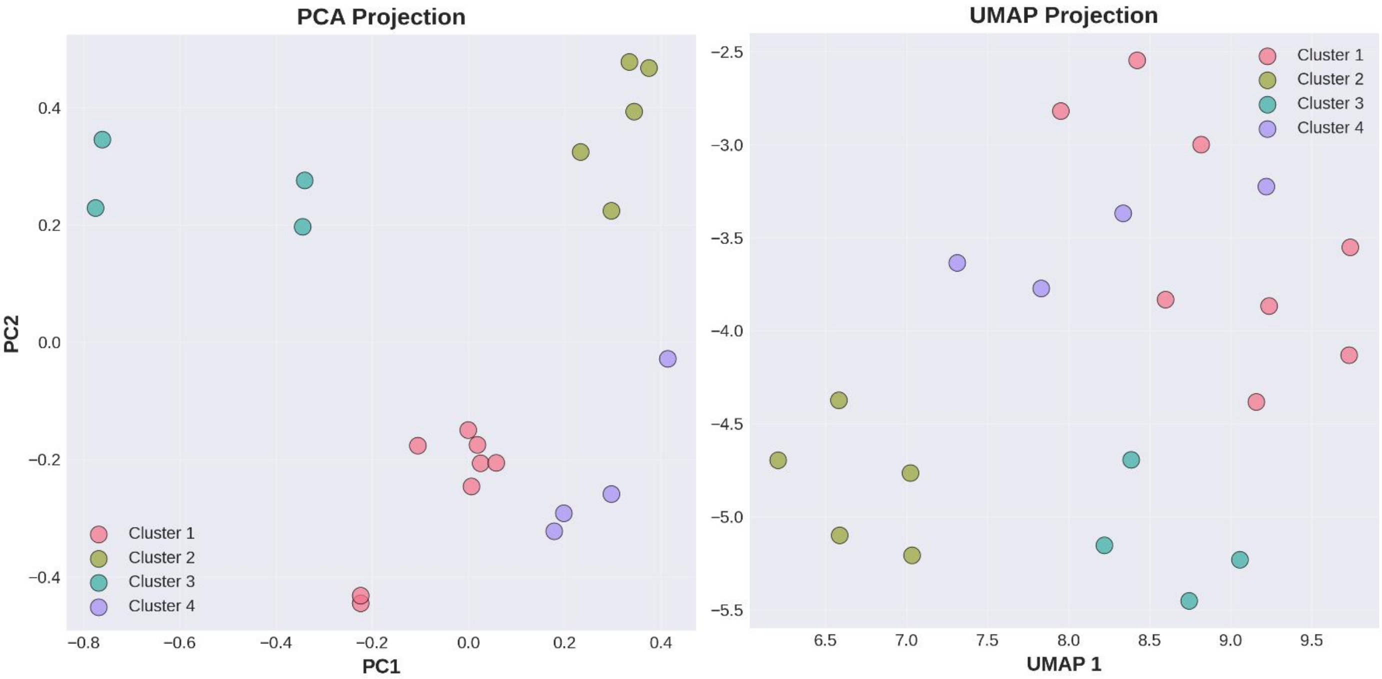

Figure S2. Embedding-based clustering of 21 representative serpin proteins using ProtT5. (a) PCA projection shows global relationships among sequences (b) UMAP projection provides a robust visualization of functional groupings and places LjSPI-1 within the parasitoid venom serpin cluster together with *P. puparum* and *C. chilonis*.

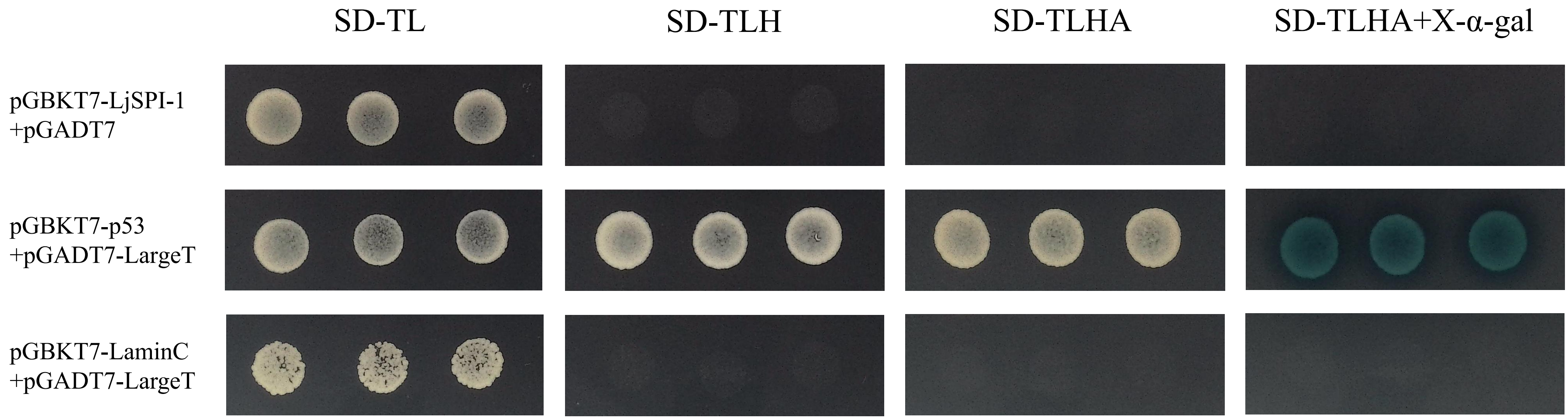

Figure S3. Autoactivation control of the bait construct pGBKT7-LjSPI-1 in yeast two-hybrid assay. Yeast strain AH109 was co-transformed with pGBKT7-LjSPI-1 + pGADT7, positive control pGBKT7-p53 + pGADT7-largeT, and negative control pGBKT7-laminC + pGADT7-largeT. Growth was tested on SD-TL (SD/-Leu/-Trp), SD-TLH (SD/-Leu/-Trp/-His), SD-TLHA (SD/-Leu/-Trp/-His/-Ade), and SD-TLHA supplemented with X-α-Gal (blue coloration indicates reporter activation). No growth or reporter activation was observed under high-stringency conditions, indicating that the bait construct does not auto-activate reporter gene expression.
